# Learning aerodynamics with neural network

**DOI:** 10.1038/s41598-022-10737-4

**Published:** 2022-04-26

**Authors:** Wenhui Peng, Yao Zhang, Eric Laurendeau, Michel C. Desmarais

**Affiliations:** 1 Department of Computer Engineering, Polytechnique Montreal, Montreal, QC Canada; 2 Department of Mechanical Engineering, Polytechnique Montreal, Montreal, QC Canada

**Keywords:** Fluid dynamics, Computer science

## Abstract

We propose a neural network (NN) architecture, the Element Spatial Convolution Neural Network (ESCNN), towards the airfoil lift coefficient prediction task. The ESCNN outperforms existing state-of-the-art NNs in terms of prediction accuracy, with two orders of less parameters. We further investigate and explain how the ESCNN succeeds in making accurate predictions with standard convolution layers. We discover that the ESCNN has the ability to extract physical patterns that emerge from aerodynamics, and such patterns are clearly reflected within a layer of the network. We show that the ESCNN is capable of learning the physical laws and equation of aerodynamics from simulation data.

## Introduction

In recent years, significant efforts have been conducted to apply NNs to fundamental science research. Two representative works revolutionized fundamental scientific research. Noe et al. created PauliNet, a deep learning method that is capable of solving Schrödinger equation^1^. Scientists from DeepMind designed the AlphaFold, a type of NN that can generate models of proteins far more accurate than any that have come before^2^. A diverse collection of intersections between NNs and physics is presented in the review paper by Carleo et al. These applications range from statistics and quantum physics to high energy and cosmology^3^.

The fluid dynamics community is no exception. The potential of using NNs to tackle fluid mechanics problems has lately been gained increasingly attention^4,5^. Jin et al. proposed NSFnets that applied NNs to solve the Navier-Stokes equations^6^. Kochkov et al. applied NNs to accelerate Computational Fluid Dynamics (CFD) simulations, and achieved a significant reduction in computation cost^7^. Li et al. further improved the computational efficiency by approximating the Navier-Stokes equations with the Fourier neural operator^8^. Once trained, these NN models can make inference within seconds and can be extremely efficient compared with traditional CFD approaches^9–15^.

However, these NN models suffer from generalization problems: their prediction accuracy drops dramatically once the parameters of the partial differential equations change^7^. To resolve this issue, Raissi et al. proposed the Physics-Informed Neural Networks (PINNs). They developed a deep learning framework for solving forward and inverse problems involving nonlinear partial differential equations^16^. In the PINN framework, prior physical knowledge is introduced as constraint, such that NNs are trained to solve supervised learning tasks while respecting the given laws of physics described by general nonlinear partial differential equations^16^. With the physical constrains, the PINNs manage to achieve state-of-the-art prediction accuracy^6,17–21^.

Despite these significant improvements towards prediction accuracy, scientists are not fully convinced with the results of NNs due to the lack of interpretability. How do PINNs solve physical problems? Do they make inference based on physical principles or just probability? Answering these questions is necessary, since people need interpretations to understand the logic driving the learned model, and make sure that the predicted results are trustworthy. However, interpreting the complex interactions between parameters and layer nodes has always been a challenging task^22–25^. This is due to the nature of NNs, also known as the ”black-box” issue: instead of solving the tasks with logical steps, NNs learn by examples and adjust parameters to improve their performance over time^26^.

Recent progress has been made to interpret how NNs solve scientific problems. In the field of mathematics, NNs have been shown to be able to aid mathematicians in discovering new conjectures and theorems^27^. In the field of chemistry, NNs have been shown to be able to capture the complex pattern of electrons moving around the nucleus^1^. In the domain of game theory, evidence has been found that human knowledge is acquired by the AlphaZero neural network as it trains on the game of chess^28^.

Is it possible that the NNs are able to develop physical insight in the process of solving physical problems, just like in other scientific domains?

Motivated by this question, in this work, we pierce the “black box” of a NN and investigate how NN solves a physical problem in aerodynamics. Our contributions are as follow: (1) we proposed a NN model that adopts a novel physics-informed structured input, the ESCNN, it outperforms existing state-of-the-art NNs in the airfoil lift coefficient prediction task. (2) We provide insight explanations as to how the ESCNN succeeds in making accurate predictions with standard convolution layers.

## Predicting lift coefficient with neural networks

Calculating the airfoil lift coefficient is one of the most critical tasks in aerodynamics. It is generally achieved by using traditional Computational Fluid Dynamics (CFD) methods, which are often known for being computational expensive. We propose a neural network based model: Element Spatial Convolution Neural Network (ESCNN), to efficiently predict the airfoil lift coefficient^29^.

**Figure 1:**
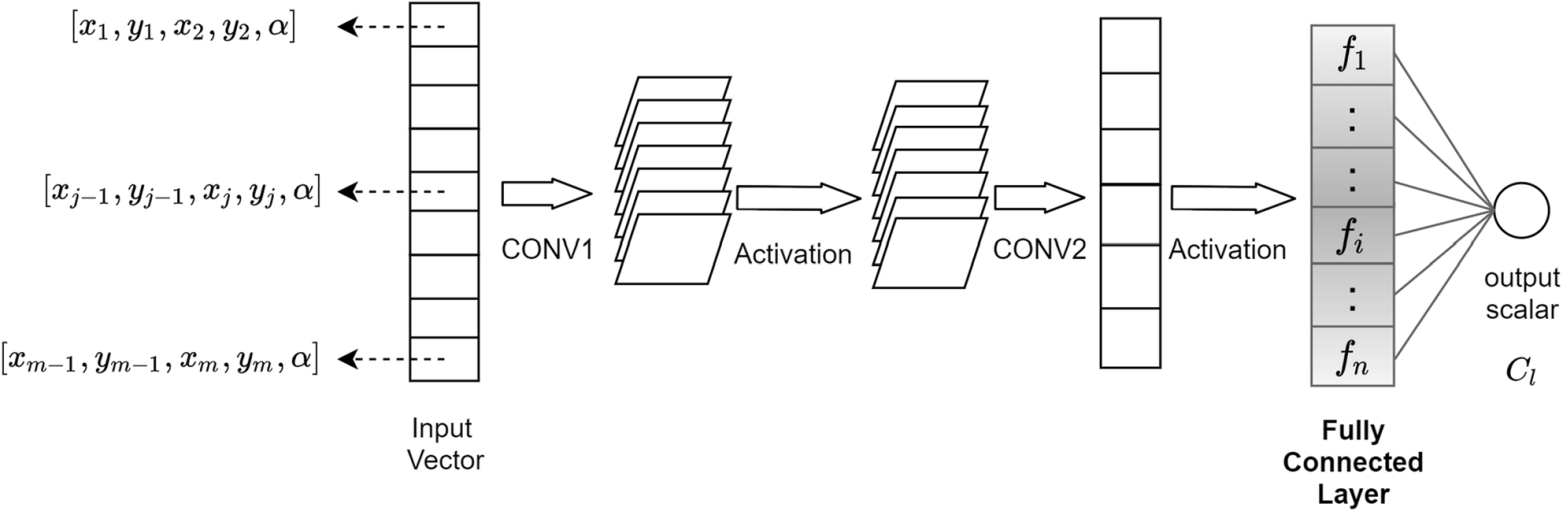
ESCNN architecture.

ESCNN is an end-to-end neural network that takes the airfoil coordinates $x_{j},y_{j}$ and angle of attack $\alpha$ as input, and gives the lift coefficient $C_{l}$ as output. The ESCNN architecture is straightforward. As shown in Fig. 1, it consists of two standard convolution layers followed by non-linear activation function, and a fully connected layer before the prediction^29^. The two convolution layers use 200 convolution filters, where the filter size of first layer is $5\times 1$ and the second layer is $1\times 1$ respectively. The last layer, fully connected layer, contains 159 neurons. Note that since the ESCNN takes sequential airfoil coordinates as input, and only convolutions and activations are involved in the architecture, therefore all the hidden layers are not permutation invariant.

The airfoil data samples are taken from the database of UIUC Applied Aerodynamic Group^30^, which covers a wide range of airfoil types from real-world designs. Each airfoil is represented by 160 points in *x*, *y* format. For the flow conditions, the Mach number *Mach* and Reynolds number *Re* are fixed to 0.3 and $1.3\times 10^7$ respectively, and the angle of attack $\alpha$ varies from $-2$ to $10^\circ$ to avoid flow separation (laminar flow). The ground truth of $C_{l}$ is computed with the CFD solver Xfoil^31^, for each angle of attack at the specified flow condition for each airfoil geometry. In total 15678 samples are generated to create the final dataset. The whole dataset is divided into training set and validation set at the ratio of 8:2. Note that in laminar flow where the dataset is generated, the lift coefficient $C_{l}$ varies linearly with the angle of attack $\alpha$, however the relationship between $C_{l}$ and the airfoil coordinates is highly-nonlinear^32^.

Table 1 shows the performance benchmarks of different models, where the regression models are provided as baselines, and three types of NNs are compared. Experiments are implemented on the Pytorch and MindSpore open-source deep learning frameworks. The Multilayer Perceptron (MLP) network model has three hidden layers, where each layer contains 256,128,128 neurons respectively. The AeroCNN is a recent NN model that achieves state-of-the-art prediction accuracy^33^. In the AeroCNN framework, the airfoils are processed as images, such that AeroCNN can adopt the typical convolution neural network architecture for image recognition^33^. The error $\varepsilon$ is the relative error defined by Eq. (1), where $\hat{C_{l}}$ denotes the predicted lift coefficient and $C_{l}$ denotes the ground truth lift coefficient.

1 $$\begin{aligned} \varepsilon =\frac{\Vert \hat{C_{l}}-C_{l}\Vert _{2}}{\Vert C_{l}\Vert _{2}}. \end{aligned}$$

**Table 1:** Performance benchmarks of different models.

Model	Parameters	Error
Linear regression	322	9.82%
MLP	961	8.43%
Multilayer perceptron	131,969	5.58%
AeroCNN^33^	266,145	3.46%
ESCNN	1561	0.97%

It is noted that even the baseline regression models can bring the error down below 10%. However, further reducing the prediction error is a difficult task: the model’s learning capacity must be large enough to accurately approximate the non-linear relationship between $C_{l}$ and airfoil coordinates. Generally, the error can be further reduced at the cost of adding more parameters to increase the model’s complexity^34^, provided sufficient training data. However, the cost becomes more significant as the error gets smaller: the parameters are doubled to reduce the error from 5.58% (Multilayer Perceptron) to 3.46% (AeroCNN).

**Figure 2:**
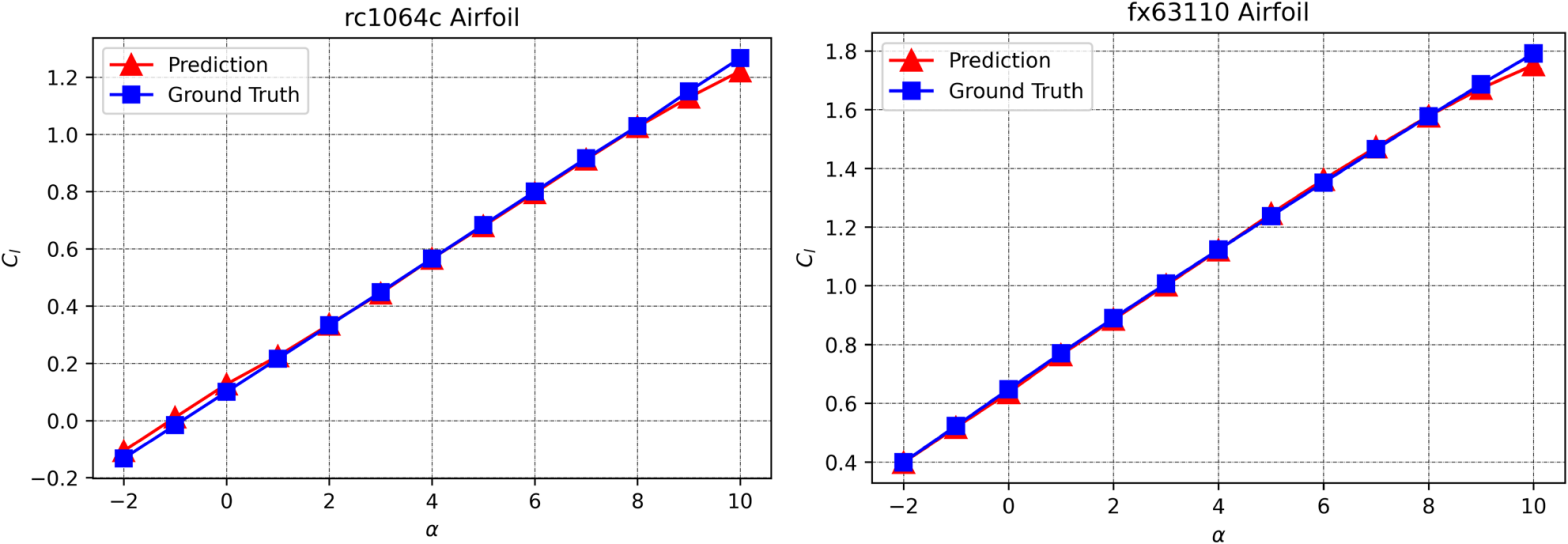
Prediction performance on validation airfoils.

In contrast, the ESCNN achieves 0.97% error with two orders of parameters less than AeroCNN. Figure 2 shows examples of the ESCNN prediction performance on the validation set, where the predictions are accurate and the $C_{l}-\alpha$ linearity is well captured. With such few parameters and limited capacity, how can ESCNN perform so well in a high-dimensional and highly-nonlinear aerodynamic system?

## Learning the Kutta condition

While investigating how ESCNN learns to make prediction, the fully connected layer, as shown in Fig. 1, draws our attention, since it is the last hidden layer before prediction.

**Figure 3:**
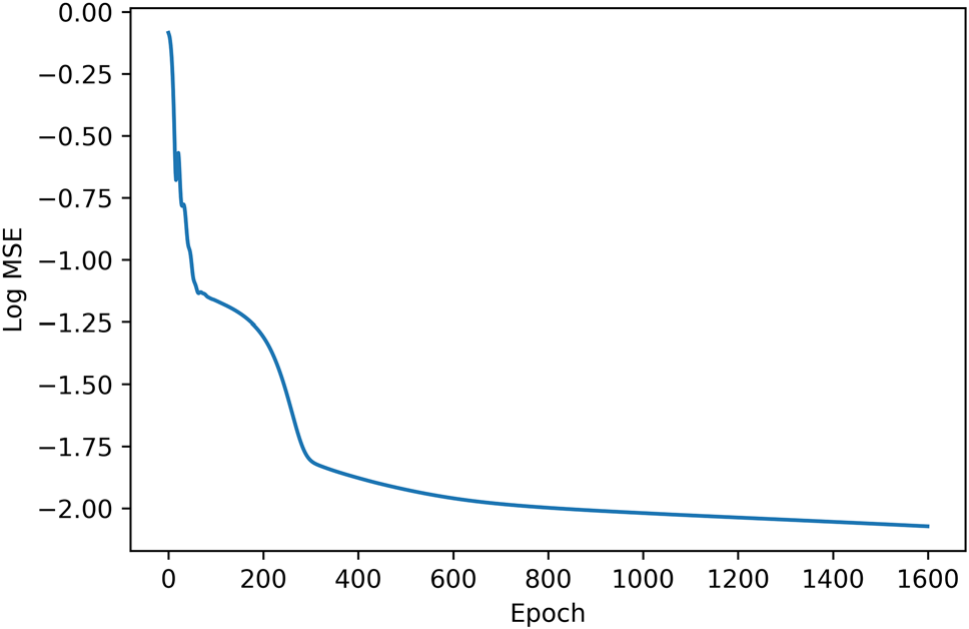
ESCNN training learning curve.

**Figure 4:**
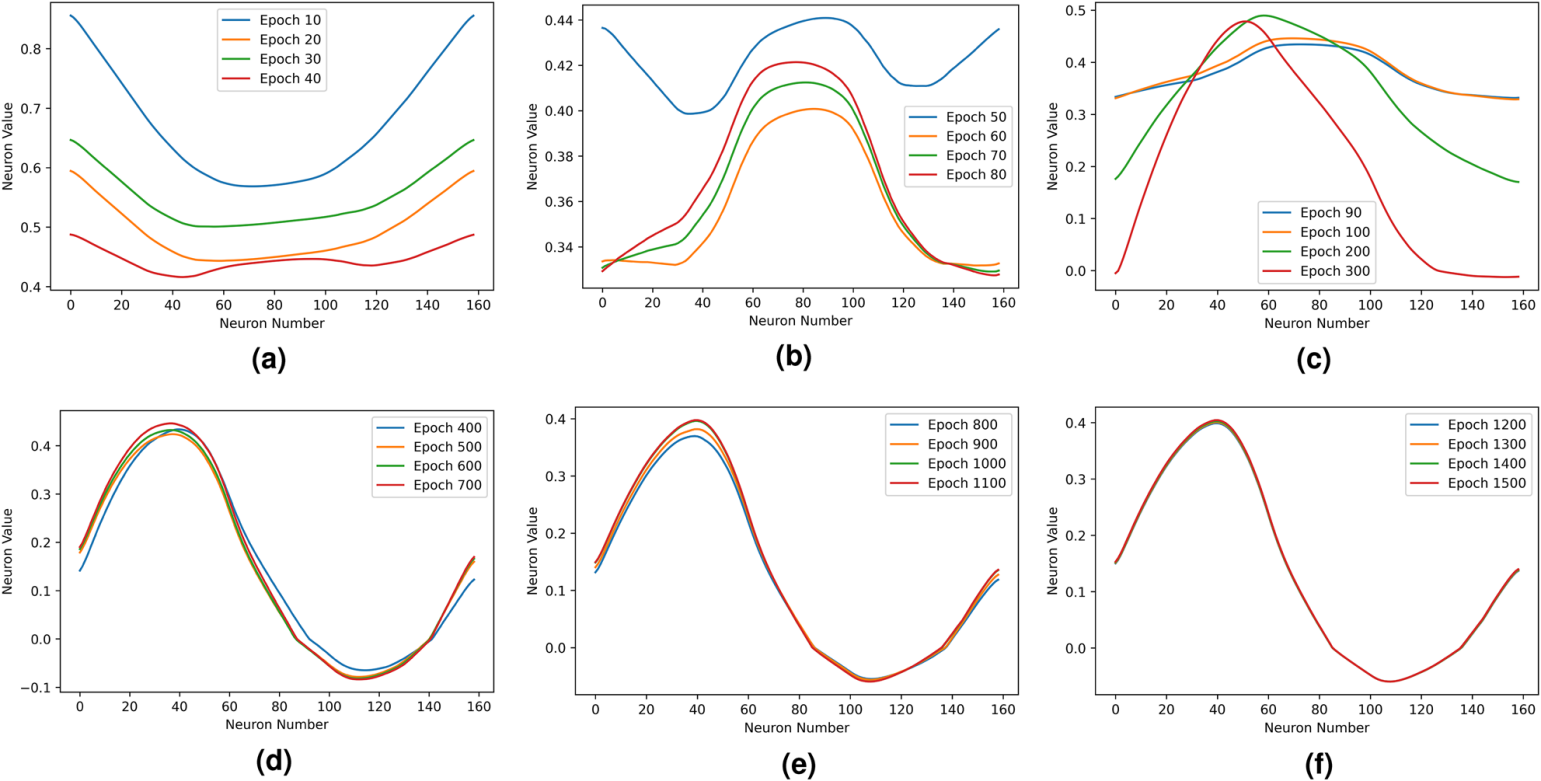
Fully connected layer evolution in training.

We track the neuron values of the fully connected layer with a test airfoil at $3^\circ$ angle of attack during the training. The test airfoil NACA 2412 contains 160 coordinates. Variations of the neuron values $[f_{1},f_{2},\ldots ,f_{n}]$ at the end of different training stages are shown in Fig. 4, where the x-axis denote the sequential index number $[1,2,\ldots ,n]$ of the neurons, and the y-axis denote corresponding neuron values $[f_{1},f_{2},\ldots ,f_{n}]$. Since the ESCNN model takes airfoil coordinates that are sequentially formatted as input, the order of coordinates is reflected in the neurons of learned hidden layers. The epoch numbers represent different training iterations, the network learns and evolves with the increasing of training epoch numbers. From the figure, it is obvious that as the training progresses, the fully connected layer is converging to a sine-shaped pattern, and this pattern begins to stabilize at around 400 epochs as shown in Fig. 4d. This result is also consistent with the learning curve that is shown in Fig. 3, as in this figure, after 400 epochs, the fitted Mean Squarer Error (MSE) converges towards a constant.

We notice an interesting phenomena, as shown in Fig. 4, the first and the last neurons values are always the same ($f_{1}$ = $f_{n}$) even at the very beginning of training. It seems like the ESCNN has learned, early in the training process, of the condition $f_{1}$ = $f_{n}$, and followed this rule strictly during the training progress. This $f_{1}$ = $f_{n}$ pattern reminds us the fundamental principle in aerodynamics: Kutta condition.

**Figure 5:**
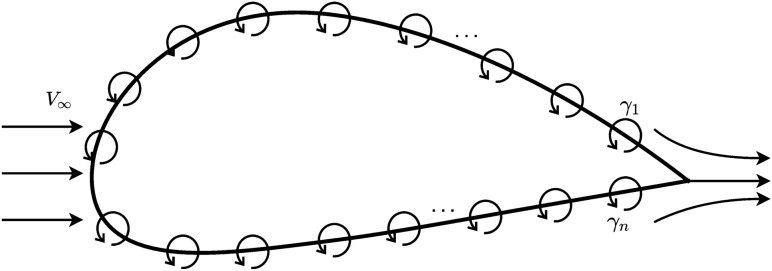
Airfoil vortex panels in free stream.

Recall the Kutta condition^32^: in fluid flow around a body with a sharp corner, the flow pattern in which fluid approaches the corner from both directions meets at the corner before flows smoothly go away from the body, as shown in Fig. 5. Equation (2) describes the Kutta condition mathematically, where $\gamma _{1}$ and $\gamma _{n}$ represents the vortex strength at the upper and lower surface of the airfoil trailing edge respectively.

2 $$\begin{aligned} \gamma _{1}=\gamma _{n}. \end{aligned}$$

Apparently, ESCNN manages to figure out the importance of the Kutta condition by itself—it prioritizes to keep the value of the first neuron and the last neuron the same during the entire learning process.

## Learning the vortex strength distribution pattern

To further investigate the meaning of the sine-shaped pattern, we compute the vortex strength distribution over airfoil using Vortex Panel Method (VPM). VPM is an engineering numerical method to compute the vortex strength distribution over airfoil. It replaces the airfoil surface with a series of vortex panels.

**Figure 6:**
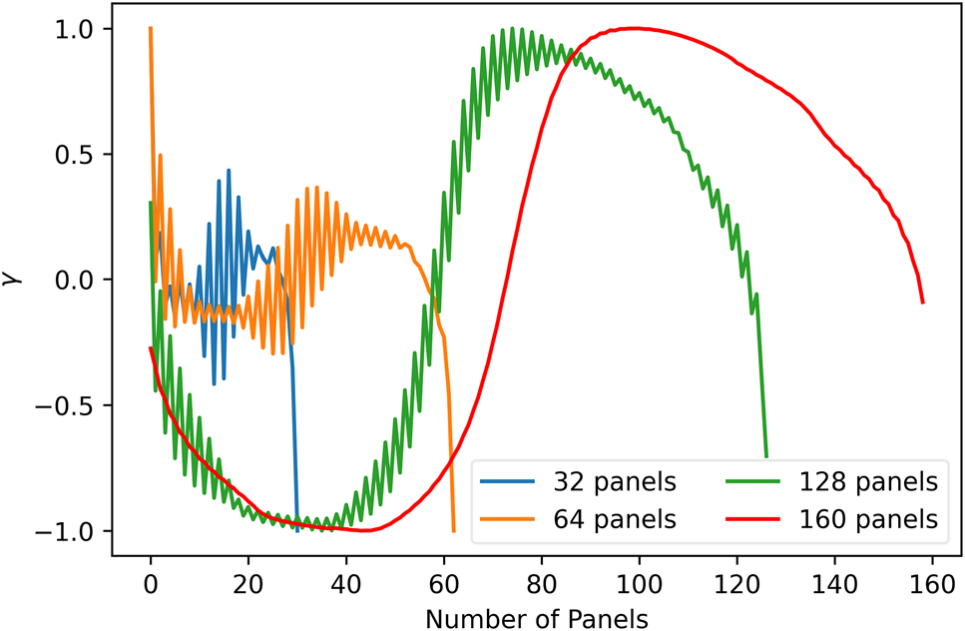
Computed vortex distribution as a function of the number of panels.

Figure 6 shows the scaled vortex strength distribution calculated by VPM over the test case NACA 2412 airfoil at $3^\circ$ angle of attack. As shown in this figure, when fewer panels are used in the calculation, the distributions of vortex strength , $\gamma$, are not smooth. The oscillations from one panel to another is a well-known flaw of VPM which is triggered by the numerical inaccuracy^32^. With the larger number of panels used during the calculation, the oscillations fades away, the results get more accurate, and the sine-shaped pattern of vortex distribution gradually emerges.

**Figure 7:**
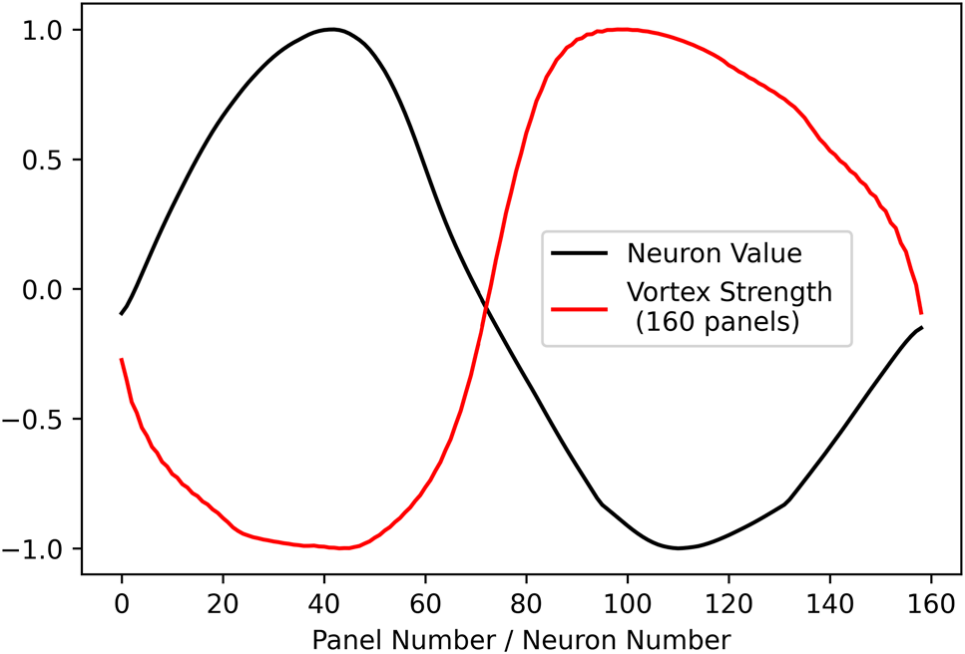
Comparison of the converged layer pattern with 160 panels vortex strength distribution.

**Figure 8:**
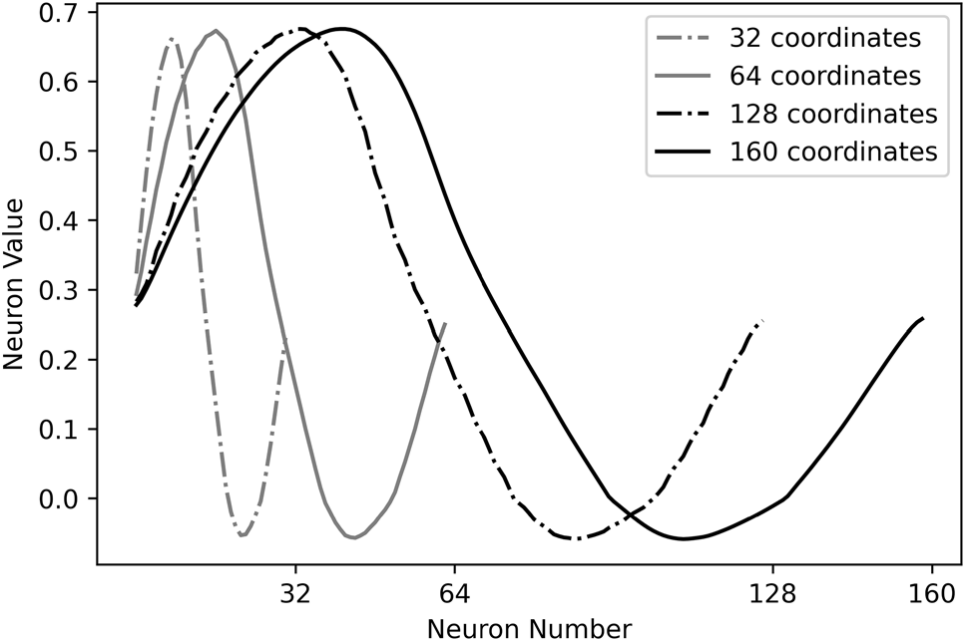
Neuron values of the fully connected layer with different number of airfoil coordinates.

Figure 7 compares the computed 160 panels vortex strength distribution with the converged layer pattern at the same scale, both curves show a similar sine-shaped pattern, except they are symmetric in relation to the horizontal axis. Furthermore, if we down-sample the number of input airfoil coordinates to fewer dimensions, the converged sine-shaped pattern are still kept by the trained network, as shown in Fig. 8, not subject to the numerical inaccuracy of VPM.

Both the converged layer pattern and the computed VPM vortex strength distribution exhibit a similar sine-shaped wave. One possible hypothesis is that the network has learned another physical concept that is a function of the vortex strength. Although there is no aerodynamics law at the moment that theoretically addresses this sine-shaped pattern, we surmise that the distribution of vortex strength over airfoil follows a certain underlying pattern.

## Learning with ReLU activation

The initial activation function we use to train the ESCNN model is the LeakyReLU, a common choice for neural networks^35^. More importantly, the output domain of LeakyReLU activation can be both positive and negative values, so is the value of vortex strength. This feature enables ESCNN to learn the vortex related physical quantity.

However, what if the network is trained with a ReLU activation function, can it still learn the vortex related physical quantity? In this section, we intentionally limit the value range of the fully connected layer by implementing another activation—ReLU function^35^. Equations (3) and (4) describes the LeakyReLU and ReLU activation function respectively, where ${\mathbf {k}}=0.5$ is the negative slope coefficient. For any real-valued input, the LeakyReLU outputs both positive and negative values, whereas ReLU outputs non-negative values only^35^.

3 $$\begin{aligned} {\text{ LeakyReLU }}(x)= & {} \max (0, x)+{\mathbf {k}} * \min (0, x). \end{aligned}$$

4 $$\begin{aligned} {\text {ReLU}}(x)= & {} (x)^{+}=\max (0, x). \end{aligned}$$

**Figure 9:**
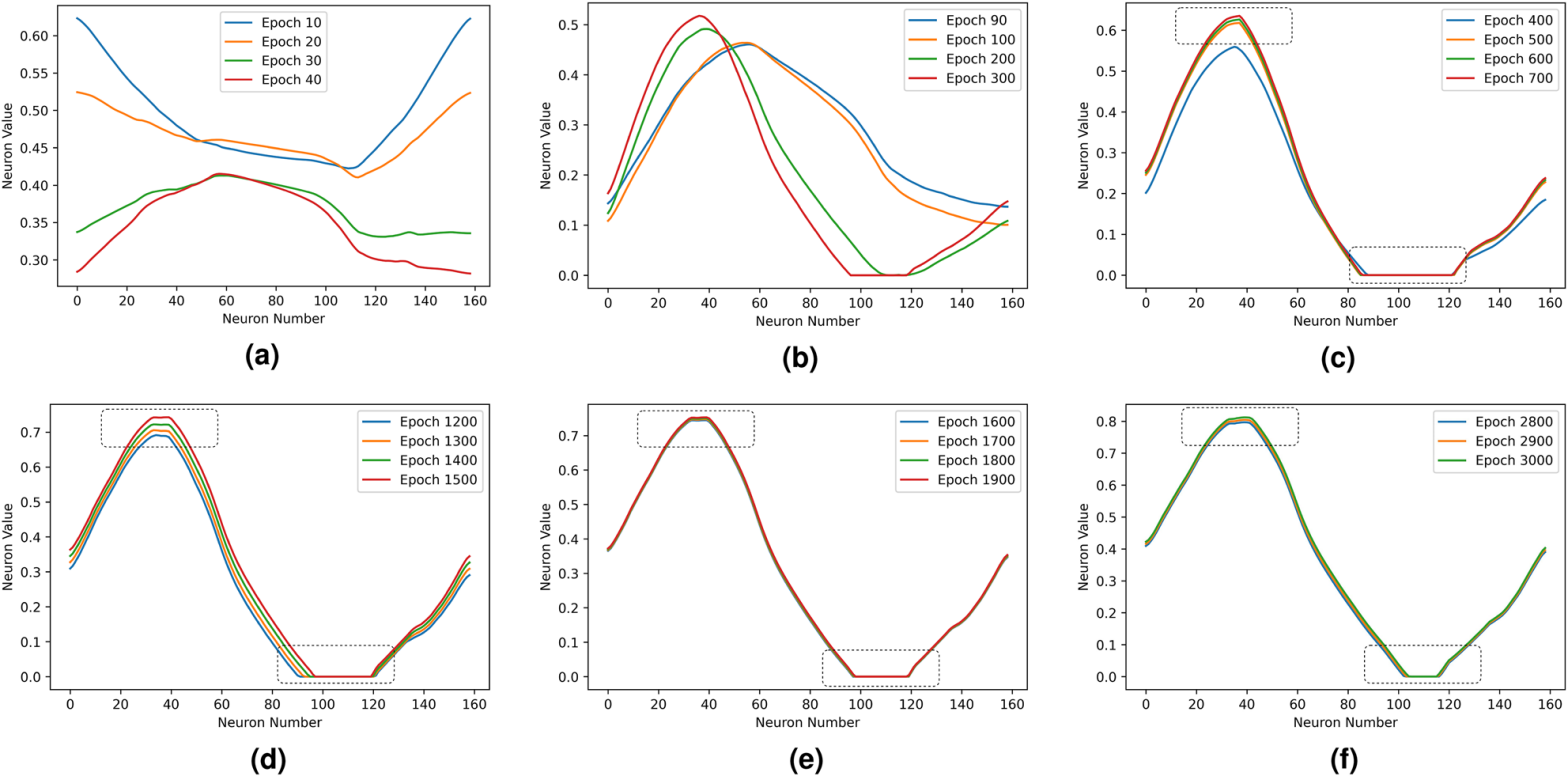
Fully connected layer under constrained activation.

Figure 9 shows that the ESCNN still learns the Kutta condition and vortex distribution pattern, although the pattern is constrained within the non-negative range. More interestingly, the fully connected layer is forcing itself to evolve into a symmetric pattern as the training goes on, as marked in the dotted rectangular in Fig. 9. Despite that there is no constraint placed in the positive domain, the evolution to such symmetric pattern is quite clear in both Figs. 4 and 9.

## Learning the lift coefficient equation

The ESCNN contains standard convolution layers, how can it outperform existing neural networks with significantly less parameters? The key reason of success is that the ESCNN makes prediction by learning the lift coefficient equation of aerodynamics at the last layer. For an airfoil at a fixed angle of attack, the lift coefficient $C_{l}$ is given by Eq. (5), where *c* is a constant^32^, $\gamma _{i}$ is the vortex strength at the panel *i*, and $l_{i}$ is the length of the panel *i*. Meanwhile, the prediction target $\hat{C_{l}}$ is obtained at the last layer of ESCNN (fully connected layer), as described by Eq. (6), where $f_{i}$ represents the neuron values and $w_{i}$ represents the learned weight parameters.

5 $$\begin{aligned} C_{l}= & {} c\sum _{i=1}^{n}\gamma _{i}l_{i}. \end{aligned}$$

6 $$\begin{aligned} \hat{C_{l}}= & {} \sum _{i=1}^{n}f_{i}w_{i}. \end{aligned}$$

We show that the ESCNN model learns Eq. (5) by matching the neuron values $[f_{1},f_{2},\ldots ,f_{n}]$ with the vortex strength over airfoil $[\gamma _{1},\gamma _{2},\ldots ,\gamma _{n}]$, and matching the learned weights $[w_{1},w_{2},\ldots ,w_{n}]$ with the length of each panels $[l_{1},l_{2},\ldots ,l_{n}]$.

We calculate the correlation coefficient between the neuron values $[f_{1},f_{2},\ldots ,f_{n}]$ and the vortex strength over airfoil $[\gamma _{1},\gamma _{2},\ldots ,\gamma _{n}]$, for each sample in the testing dataset. The correlation coefficient *cov* is defined by Eq. (7), where ${\mathbf {x}}$ and ${\mathbf {y}}$ are both vectors. Results show that the neuron values and vortex strength of all testing samples are highly correlated: the average of correlation coefficient is $-0.975$, with a standard deviation of $6.4\times 10^{-3}$, an example is shown in Fig. 7.

7 $$\begin{aligned} cov = \frac{{\mathbf {x}} \cdot {\mathbf {y}}}{\Vert {\mathbf {x}}\Vert \Vert {\mathbf {y}}\Vert }. \end{aligned}$$

**Figure 10:**
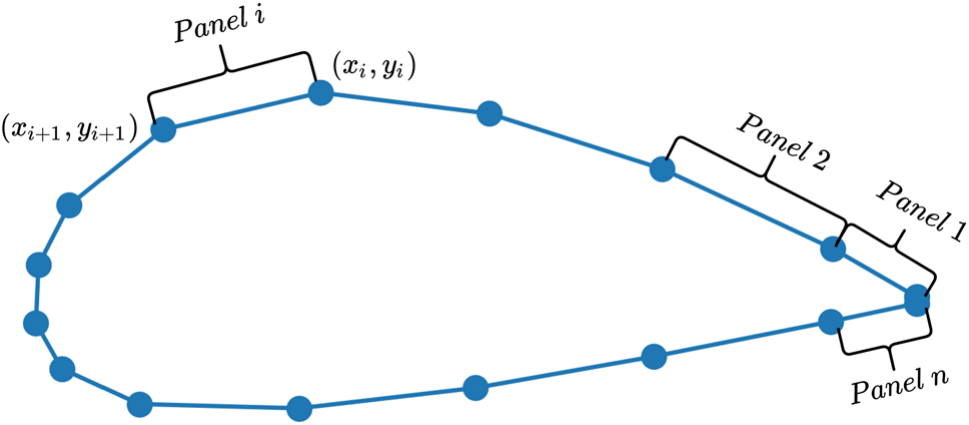
Panel distribution over the airfoil surface.

**Figure 11:**
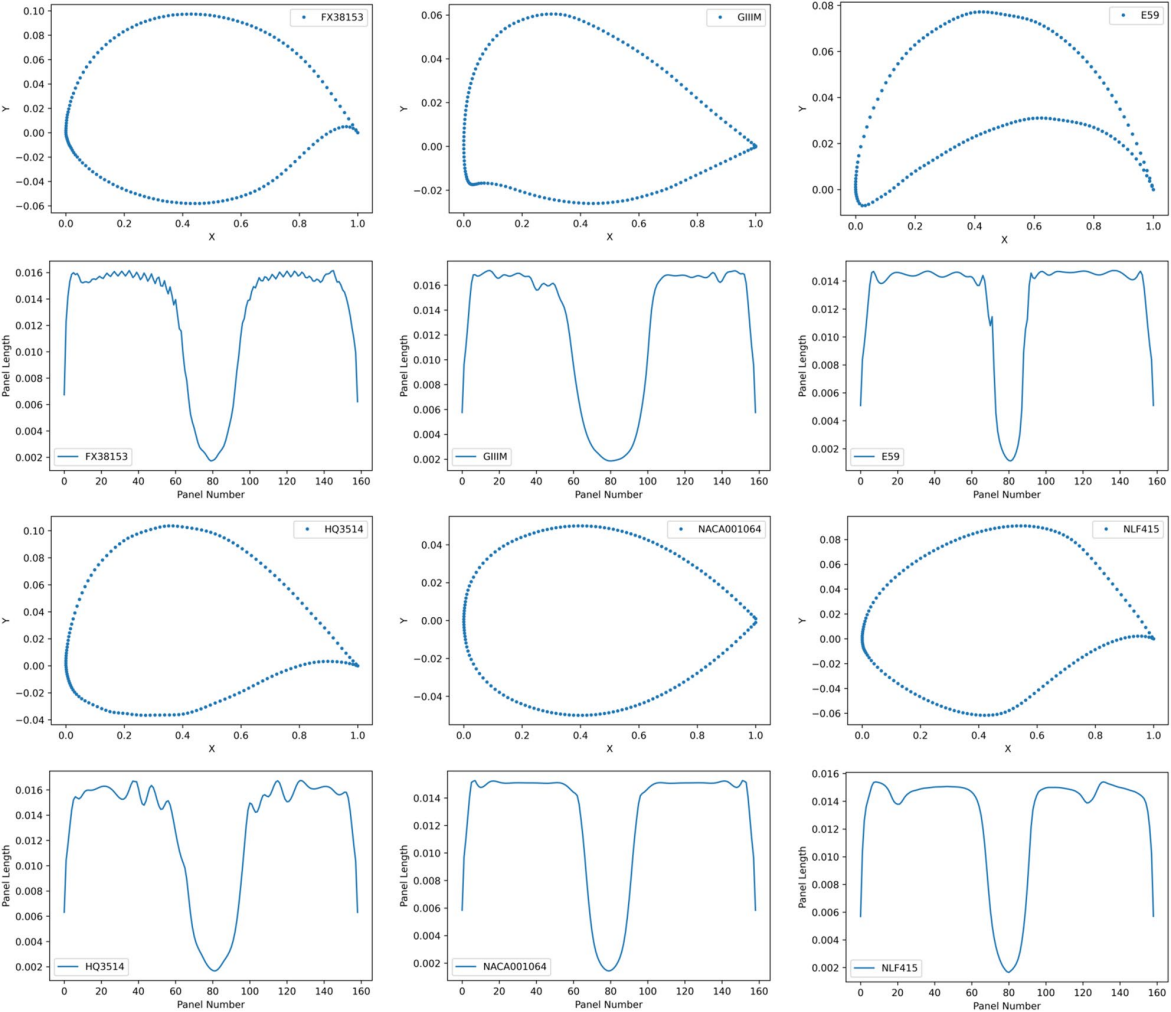
Randomly sampled airfoils and their panel length distribution.

We also calculate the correlation coefficient between the length of each panels $[l_{1},l_{2},\ldots ,l_{n}]$ and the learned weights $[w_{1},w_{2},\ldots ,w_{n}]$. Note that the learned weights are fixed after training, however, the length of each panels $[l_{1},l_{2},\ldots ,l_{n}]$ varies across different airfoils. The panels of an airfoil refer to the segments between two sequential points, as shown in Fig. 10. The panels are serially numbered from 1 to *n* according to their locations on the airfoil surface (starting from the trailing edge, along the upper surface to the leading edge and back around the lower surface to trailing edge). The length of each panel $l_{i}$ is the spatial distance between two sequential coordinates as defined by Eq. (8). Figure 11 shows the randomly sampled airfoils, and their corresponding panel length distribution.

**Figure 12:**
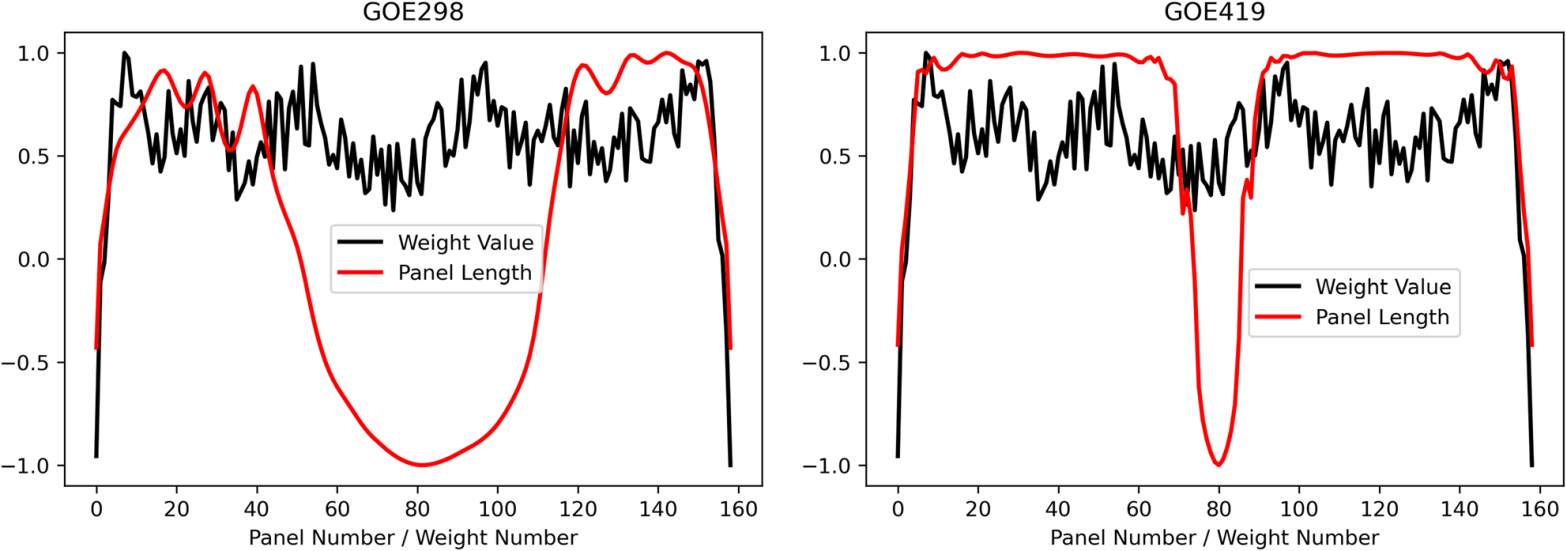
Comparison of learned weights and panel length distribution at the same scale.

Figure 12 compares the learned weights and the panel length distribution of example airfoils at the same scale. Despite that panel length distributions are different across airfoils, they are close to symmetric. This symmetric pattern explains why the fully connected layer evolves for symmetry during training as shown in Figs. 4 and 9. The learned weights are not as highly correlated with the panel length distribution, as compared with the correlation between neuron values and vortex strength. However, the symmetric pattern of panel length distribution has been captured by the learned weights. Moreover, we notice from Table 2 that the correlation between the panel length and learned weights affects the prediction performance: higher correlation leads to lower prediction error. Different airfoils have different panel length distributions, whereas the learned weights are fixed, failing to match the weights for corresponding airfoil leads to the increase of prediction error.

8 $$\begin{aligned} l_{i} = \sqrt{(x_{i+1}-x_{i})^2 + (y_{i+1}-y_{i})^2}. \end{aligned}$$

**Table 2:** Correlation (between the learned weights and the panel length) of different airfoils and prediction error.

Airfoil	Correlation	Error (%)
GOE298	0.16	1.22
GOE419	0.43	0.71
EH2012	0.32	0.93
HQ259B	0.30	0.97
NACA632615	0.27	1.03

How does ESCNN manages to learn the physical quantity of vortex strength and the panel length? The reason is that ESCNN adopts a structured input that incorporates prior physical knowledge.

In the vortex panel method, the vortex strength $\gamma _{1}$ to $\gamma _{n}$ are obtained by solving a linear system of *n* equations, and solving each equation requires the angle of attack $\alpha$ and the coordinates of corresponding panel^32^. Inspired by the vortex panel method, we combine the panel coordinates $x_{i},y_{i},x_{i+1},y_{i+1}$ and the angle of attack $\alpha$ as an element unit, and then concatenate all the element units into a single vector, as shown in Fig. 1. The first layer, CONV1, performs 1*D* convolution over each element unit, and each element unit contains sufficient information to solve the vortex strength and panel length.

The physics-informed structured input allows the convolution layer to pick up the vortex strength $\gamma _{i}$ and panel length $l_{i}$, and further allows the fully connected layer to learn the lift coefficient equation.

## Conclusions

In this work, we propose a neural network model that outperforms existing state-of-the-art NNs in the airfoil lift coefficient prediction task, with two orders of less parameters. We further investigate how the ESCNN makes accurate predictions.

The ESCNN network learns to constrain the first and last neurons to be equal, during the entire learning process. This is the evidence that it self-learns the fundamental aerodynamics principle, the Kutta condition. Moreover, the fully connected layer converges to a sine-shaped wave pattern that is highly correlated to the vortex strength distribution over airfoil, which demonstrates that the network learns the vortex related physical quantity. In addition, we explore ESCNN’s learning ability with constrained activation by replacing LeakyReLU to ReLU function. The results show that even with a limited value range of the neurons, ESCNN can still learn the critical physics of Kutta condition and the vortex distribution pattern. In the end, we show that the network learns lift coefficient equation at the last layer.
